# Functional nanostructure-loaded three-dimensional graphene foam as a non-enzymatic electrochemical sensor for reagentless glucose detection†

**DOI:** 10.1039/d0ra05553k

**Published:** 2020-09-11

**Authors:** Qianshi Liu, Huage Zhong, Miao Chen, Chang Zhao, Yan Liu, Fengna Xi, Tao Luo

**Affiliations:** a Guangxi Medical University Cancer Hospital 71 Hedi Road Nanning 530021 PR China lt_non_stop@163.com luotao@gxmu.edu.cn; b Department of Chemistry, Zhejiang Sci-Tech University 928 Second Avenue, Xiasha Higher Education Zone Hangzhou 310018 PR China fengnaxi@zstu.edu.cn

## Abstract

Non-enzymatic and reagentless electrochemical sensors for convenient and sensitive detection of glucose are highly desirable for prevention, diagnosis and treatment of diabetes owing to their unique merits of simplicity and easy operation. Facile fabrication of a three-dimensional (3D) sensing interface with non-enzymatic recognition groups and an immobilized electrochemical probe remains challenge. Herein, a novel non-enzymatic electrochemical sensor was developed for the sensitive and reagentless detection of glucose by loading functional nanostructure on 3D graphene. Monolithic and macroporous 3D graphene (3DG) foam grown by chemical vapor deposition (CVD) served as the electrode scaffold. Prussian blue (PB) and gold nanoparticles (AuNPs) were first co-electrodeposited on 3DG (3DG/PB-AuNPs) as immobilized signal indicator and electron conductor. After a polydopamine (PDA) layer was introduced on 3DG/PB-AuNPs *via* facile self-polymerization of dopamine to stabilize internal PB probes and offer chemical reducibility, the second layer of AuNPs was *in situ* formed to assemble the recognition ligand, mercaptobenzoboric acid (MPBA). Owing to the high stability of PB and good affinity between MPBA and glucose, the non-enzymatic sensor was able to be used in reagentless detection of glucose with high selectivity, wide linear range (5 μM–65 μM) and low detection limit (1.5 μM). Furthermore, the sensor was used for the detection of glucose level in human serum samples.

## Abstract 2

A non-enzymatic electrochemical sensor was fabricated by loading functional nanostructure on three-dimensional graphene foam for reagentless detection of glucose with high sensitivity and stability.

## Graphical abstract

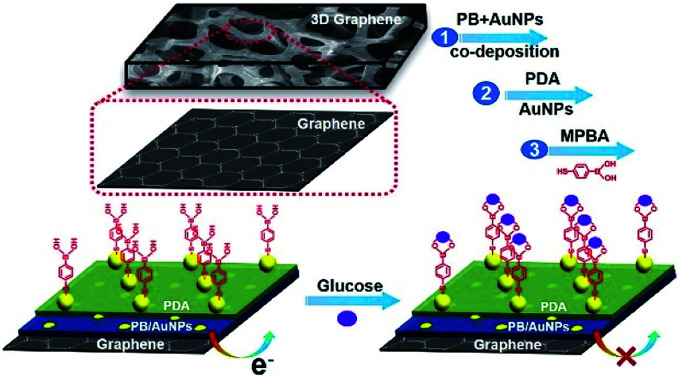

## 1. Introduction

Diabetes is related to a glucose metabolic disorder arising from insulin deficiency or resistance, which has become the second killer among modern diseases owing to serious complications (*e.g.* high blood pressure, blindness, heart disease, and kidney failure).^1–3^ A close monitoring of blood glucose concentration is of great significance for prevention, diagnosis and treatment of diabetes. In addition, detection of glucose is also crucial in fields of biotechnology and food industry. Tremendous efforts have been put into the development of sensitive, selective, and reliable methods to detect blood glucose. Among various developed techniques (*e.g.*, spectrophotometry, fluorescence, chemiluminescence), electrochemical sensors have received considerable attention due to their unique merits of simplicity, low cost, high sensitivity, simple instrumentation, easy operation, and ease of integration.^4–8^ For most glucose electrochemical sensors, the detection is mediated by enzymes (*e.g.* glucose oxidase), which lead to glucose oxidation to hydrogen peroxide (H_2_O_2_) and gluconolactone, and the concentration of H_2_O_2_ can be measured electrochemically.^9–11^ In spite of their wide application, enzyme-based biosensor systems suffer from low long-term stability, poor tolerance in experimental conditions and high cost.^12–21^ Therefore, there is a significant interest in the development of non-enzymatic and sensitive glucose sensors.

As a core component of the electrochemical sensor, electrode material and architecture are critical to ensure sensing performance.^22–24^ Owing to fascinating properties including high specific surface area, excellent electronic transport properties, and outstanding electrocatalytic activity (due to, *e.g.*, defects, edge sites, oxygenated functional groups), graphene has been widely employed in electrochemical sensing.^25–29^ In addition to two-dimensional (2D) graphene nanosheet, three-dimensional graphene (3DG) foam prepared by chemical vapor deposition (CVD) method is ideal to serve as the freestanding electrode for the fabrication of high-performance electrochemical sensors owing to its superior characteristics including large pore structure, high surface area, unhindered mass diffusion, high conductivity and stability.^23,30–32^ However, 3DG foam is defect-free and possesses ultrahigh hydrophobicity, which imposes difficulties in its surface modifiability and biosensing application. Therefore, it is highly desired to develop facile surface modification strategy for extending the application of 3DG in sensing and biosensing.

Recognitive ligands with high affinity towards target analytes and detection modes are also crucial for electrochemical sensors. Boronic acid (PBA) and its derivatives (*e.g.* mercaptobenzoboric acid-MPBA) present high affinity towards glucose by forming five-membered cyclic with diols, demonstrating rapid and sensitive response towards glucose. Owing to the advantages such as low cost, high stability and reversible regeneration, PBA and its derivatives exhibit great potential for the fabrication of non-enzymatic glucose sensors.^33–35^ On the other hand, reagentless electrochemical sensors which rely on the immobilization of mediator on the surface of electrode is advantages for detection as compared to those use solution-phase mediator by reducing diffusion limit and avoiding side-effects (*e.g.*, contamination to the target system).^31^

In the present work, a novel non-enzymatic and reagentless 3D electrochemical sensor based on 3D graphene is presented for glucose detection. Prussian blue, a well-known electrochemical indicator, was co-deposited with Au nanoparticles (AuNPs) on 3DG electrode by a simple one-step electrodeposition (3DG/PB-AuNPs). To improve the stability of immobilized electrochemical probe, polydopamine (PDA) layer was further grown through self-polymerization of dopamine (DA). Then, the second layer of AuNPs were *in situ* formed owing to the reduction ability of PDA and applied for the assembly of mercaptobenzoboric acid (MPBA) by forming Au–S bond. When glucose bound with MPBA, electrochemical signal of PB on electrode surface decreased, which linearly correlated with the concentration of glucose. The herein demonstrated method to functionalize 3DG and fabricate non-enzymatic sensor is simple and easily operated. The obtained electrochemical sensor exhibits high sensitivity and good stability owing to facilitated electronic transport of PB by AuNPs, improved stability of PB by PDA layer, high affinity between MPBA and glucose, good conductivity, and high surface area of 3D graphene. In comparison with the previous non-enzymatic sensors, we show for the first time the non-enzymatic and reagentless detection of glucose based on 3D graphene. As 3D graphene is now commercially available, this strategy might open up a new venue for the functionalization of 3D graphene and be extended for the development of various highly sensitive and reagentless 3D sensors.

## 2. Experimental

### 2.1 Preparation of 3D non-enzymatic electrode

As previously described,^31,36^ 3D porous graphene was synthesized by CVD method using porous nickel film as the growth substrate. Free-standing 3D porous graphene was obtained after removal of nickel by hydrochloric acid (24 h, 80 °C). 3DG electrode was prepared by fixing 3DG (0.5 cm × 0.5 cm, 1 mm thick) onto a glass slide. Electrical lead was made by silver paint and copper wire insulated with silicone rubber.

For the electrochemical co-deposition of PB and AuNPs, 3DG electrode was immersed in a mixed solution (pH 3.2) containing KNO_3_ (0.1 mM), K_3_[Fe(CN)_6_] (1 mM) and HAuCl_4_ (1 mM) and scanned for 30 cycles with a potential window of 0–1 V. The obtained 3DG/PB-AuNPs electrode was then immersed in DA solution (0.38 mg ml^−1^, 0.1 M phosphate buffer solution-PBS, pH 8.5) for 1 h to form a PDA layer (3DG/PB-AuNPs/PDA). To further deposit AuNPs, 3DG/PB-AuNPs/PDA was immersed in HAuCl_4_ solution (2 mM) with slight stirring at room temperature for 2 h. The obtained 3DG/PB-AuNPs/PDA-AuNPs was immersed in MPBA solution (0.77 mg ml^−1^) at room temperature for 4 h to assemble MPBA on AuNPs (3DG/PB-AuNPs/PDA-AuNPs/MPBA).

### 2.2 Characterizations

Scanning electron microscopy (SEM) was conducted on a field-emission scanning electron microscopy (S-4800, Hitachi, Japan) operated at 3 kV. Element mapping images were obtained using the energy dispersive spectrum (EDS) equipped on SEM. Raman spectrum were recorded with laser excitation wavelength of 514 nm on CRM200 Raman System (WITeck, Germany). Electrochemical measurements (cyclic voltammetry-CV and differential pulse voltammetry-DPV) were performed on a CHI 660D electrochemical analyzer (Shanghai CH Instruments, China). A conventional three-electrode system was used with a bare or modified 3DG electrode as the working electrode, Ag/AgCl electrode (saturated with KCl) as the reference electrode, and platinum disk (1 cm × 1 cm) as the auxiliary electrode. KNO_3_ solution (0.1 M) was used as the electrolyte solution.

### 2.3 Electrochemical detection of glucose

3DG/PB-AuNPs/PDA-AuNPs/MPBA electrode was immersed into different concentration of glucose solution (0.05 M PBS, pH 7) for 15 min. Then the change of DPV signal of the electrode before and after glucose binding was measured.

## 3. Results and discussion

### 3.1 Characterizations of 3DG electrode

**Fig. 1:**
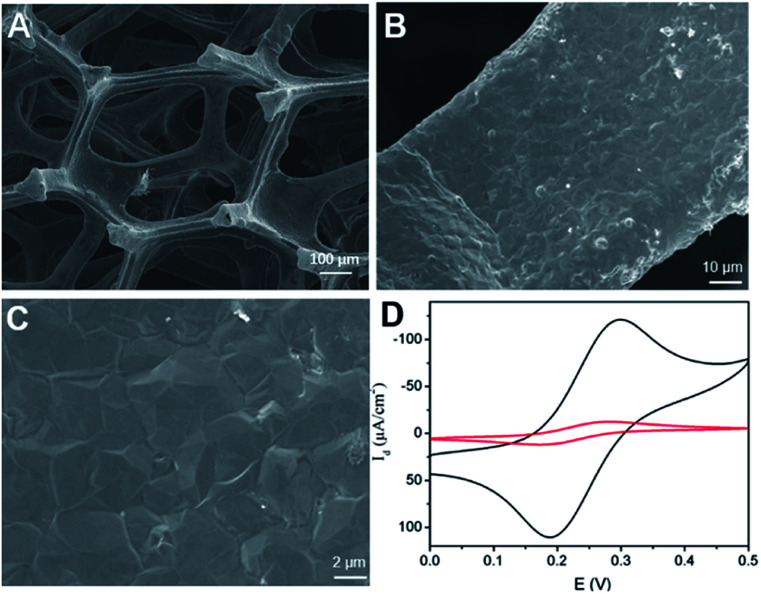
(A–C) SEM images of 3DG at different magnifications. (D) CVs obtained on GCE or 3DG electrode in 0.1 M KNO_3_ containing 1 mM [Fe(CN)_6_]^4−^/^3−^.

Scanning electron microscope (SEM) is used to characterize the morphology of 3DG electrode. Fig. 1A–C demonstrated SEM images of 3DG grown by CVD at different magnifications. It can be seen that 3DG has a monolithic structure with penetrating porous structure and smooth surface. The wrinkle of graphene could be seen in SEM image at high magnification. Raman features of 3DG is also investigated. As displayed in Fig. S1A (ESI),† characteristic G band located at ∼1580 cm^−1^ is observed, that represents the E_2g_ phonon of sp^2^ bonds of carbon atoms. The absence of defective D band (usually at ∼1361 cm^−1^) evidences the defect-free graphene structure in 3DG. The energy dispersive spectrum (EDS) reveals the high content of carbon (Fig. S1B in ESI†). As the intrinsic graphene structure has been proved by Raman spectroscopy, a small amount of oxygen may come from O_2_ or H_2_O in the atmosphere that can form chemical bonding (*e.g.* C–OH or C–O–C) on the graphene structure (The nitrogen signal is used as a reference for subsequent modification). The electrochemical performance of 3DG electrode was investigated by cyclic voltammetry (CV) measurement of the standard redox probe (Fe(CN)_6_^3−^/Fe(CN)_6_^4−^). As shown in Fig. 1D, the redox peak potentials of Fe(CN)_6_^3−^ on 3DG electrode are approximately the same as that on glassy carbon electrode (GCE), the most commonly used 2D carbon electrode. In comparison to GCE, 3DG electrode gives remarkably higher current density owing to its good conductivity, high active surface, and unhindered diffusion.

### 3.2 Preparation of 3D non-enzymatic electrode

**Fig. 2:**
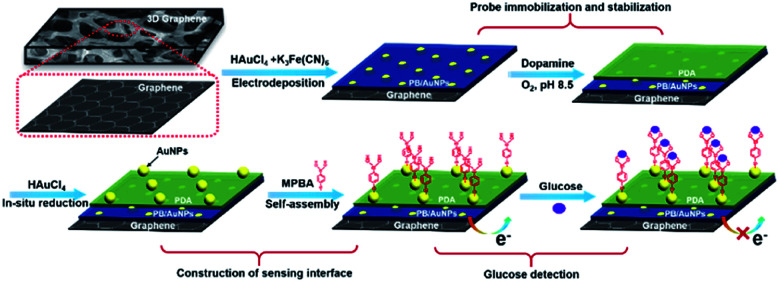
Schematic illustration for the fabrication of 3D non-enzymatic and reagentless glucose sensor.

Immobilization of electrochemical mediator and introduction of recognitive group on electrode are two key elements for the construction of non-enzymatic and reagentless glucose sensor. As illustrated in Fig. 2, Prussian Blue (PB), a well-known electrochemical inorganic mediator, is chosen owing to excellent redox electrochemistry, simple preparation, and low cost. However, PB-based electrochemical biosensors suffer from low electron conductivity and poor stability. In this contribution, highly conductive PB is easily immobilized on 3DG electrode through co-deposition with AuNPs. At the same time, to improve the stability of the deposited PB, polydopamine (PDA) layer was then formed on 3DG/PB-AuNPs through self-polymerization of dopamine (DA) under alkaline conditions. Using the reduction ability of PDA, AuNPs could be *in situ* formed on PDA layer, which can further immobilize MPBA *via* Au–S bind for specific binding of glucose.

**Fig. 3:**
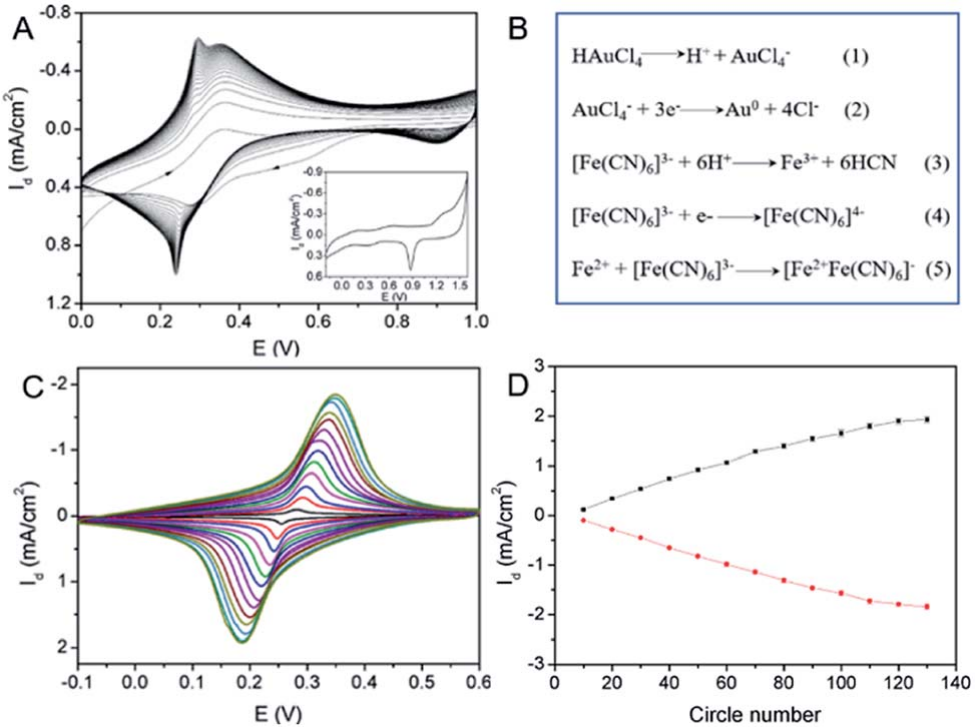
(A) CVs obtained on 3DG electrode in continuous scan in the mixture solution of KNO_3_, K_3_[Fe(CN)_6_] and HAuCl_4_. Inset was CV obtained on 3DG/PB-AuNPs electrode in H_2_SO_4_ medium. (B) The possible mechanism for the co-deposition of PB-AuNPs. (C) CVs obtained on 3DG/PB-AuNPs electrode prepared using electrodeposition at different scan cycles (10–130 cycles) in KNO_3_ solution. (D) Dependence of current density on scan circle number in electrodeposition.

**Fig. 4:**
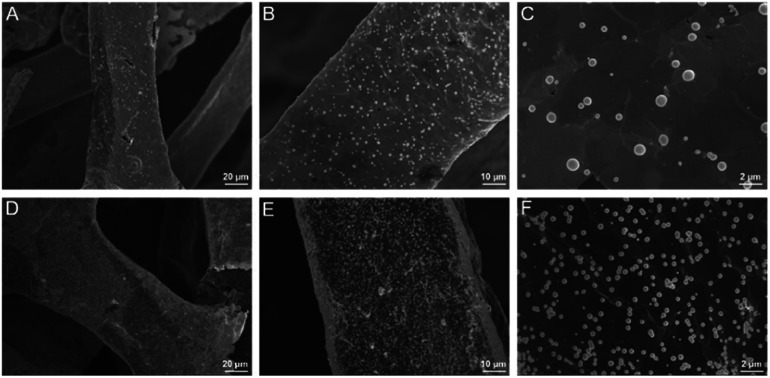
SEM images of 3DG/PB-AuNPs (A–C) and 3DG/PB-AuNPs/PDA-AuNPs (D–F) at different magnifications.

Electrochemical co-deposition of PB and AuNPs was carried out in acidic mixture solution containing KNO_3_, K_3_[Fe(CN)_6_] and HAuCl_4_. When CV scan was performed between 0–1.0 V, a redox pair with continuous increasing peaks is observed (Fig. 3A), which were the typical feature of PB modified electrodes from conversion of PB (K[Fe^3+^Fe^2+^(CN)_6_]) and Prussian white (PW, K_2_[Fe^2+^Fe^2+^(CN)_6_]).^15–19^ The obtained electrode also exhibits characteristic peaks of Au when scanned in sulfuric acid, indicating co-deposition of PB and AuNPs. The possible mechanism for the formation of PB-AuNPs is given in Fig. 3B. In the electrochemical scan process, AuCl_4_^−^ is electrochemically reduced to Au^0^ and Fe(CN)_6_^3−^ was reduced to Fe(CN)_6_^4−^. At the same time, Fe(CN)_6_^3−^ dissociated to Fe^3+^ at acid medium. The reaction between Fe(CN)_6_^4−^ and Fe^3+^ resulted in PB ([Fe^3+^Fe^2+^(CN)_6_]^+^). In order to determine the optimal electrodeposition conditions, redox characteristics of 3DG/Au-PB electrode prepared using different CV scan cycles was investigated. As shown in Fig. 3C and D, the initial increasement of deposition cycles increased the peak current. However, the higher the scan cycle, the larger peak-to-peak potential and the longer the electrodeposition time. Thus, 30 cycles of CV scan were chosen for further investigation. The formation of PB-AuNPs nanocomposites on 3DG electrode surface was also revealed through SEM investigation. As shown in Fig. 4A–C, deposited nanoparticles can be clearly seen on the surface of 3DG. At the same time, this modification did not significantly change the morphology of 3DG and flake-like graphene structure can also be seen. Characteristic signals of N (1.3%) and Fe (0.4%) atoms form PB (K[Fe^3+^Fe^2+^(CN)_6_]), and Au atoms (1.6%) from Au nanoparticles were revealed by element mapping images of EDS, confirming the formation of PB-AuNPs layer (Fig. S2 in ESI†).

Inspired by mussel adhesive proteins, PDA is well known as one of the simplest and most versatile substances to functionalize materials and displays excellent biocompatibility.^37–40^ Using simple self-polymerization of DA, PDA could be easily prepared on 3DG/PB-AuNPs. As displayed in Fig. S3 in ESI,† the contents of O and N atoms increased resulting from the PDA modification. In addition, it is worth noting that Fe and Au signals are not detected on 3DG/PB-AuNPs/PDA, which proved the protective effect of PDA layer on PB. The obtained 3DG/PB-AuNPs/PDA was further functionalized by AuNPs formed through *in situ* reduction of AuCl_4_^−^ by PDA. As demonstrated in Fig. 4D–F, AuNPs uniformly cover the electrode surface. The presence of characteristic signals of Au atoms (1.7%) are also revealed by element mapping image of EDS (Fig. S4 in ESI†). These *in situ* grown AuNPs can not only increase the electron transfer rate, but also can be used as sites to introduce recognition ligands, mercaptobenzoboric acid (MPBA), through interaction between AuNPs and –SH group of MPBA.

**Fig. 5:**
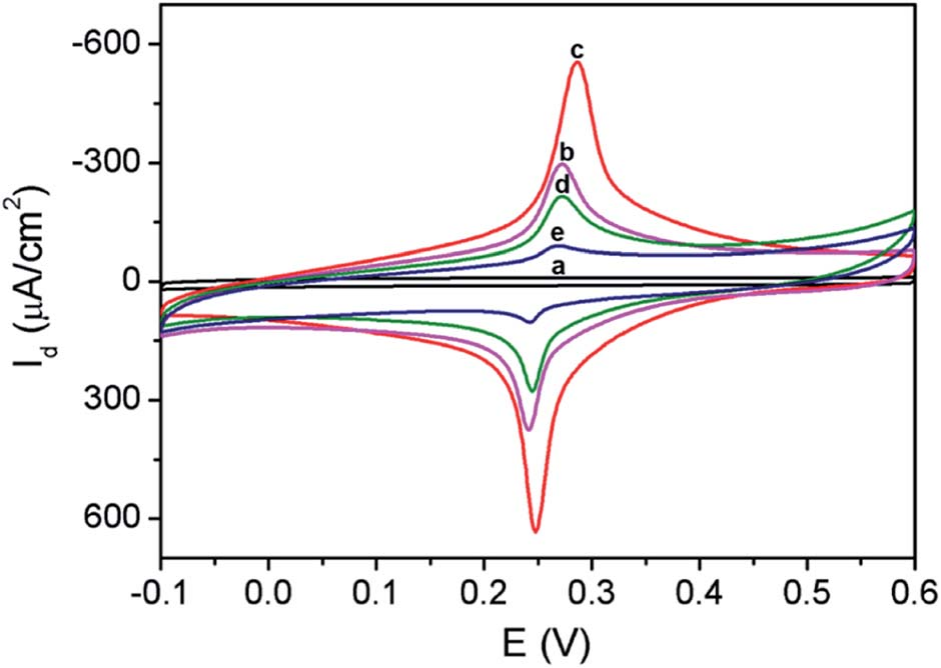
CVs obtained on (a) 3DG, (b) 3DG/PB-AuNPs, (c) 3DG/PB-AuNPs/PDA-AuNPs, (d) 3DG/PB-AuNPs/PDA-AuNPs/MPBA in 0.1 M KNO_3_ solution. (e) CV obtained on 3DG/PB-AuNPs/PDA-AuNPs/MPBA in 0.1 M KNO_3_ solution containing 10 μM glucose.

The process for the construction of enzyme-free sensor was also characterized based on electrochemical signal of immobilized PB indicator. As shown in Fig. 5, 3DG electrode exhibits almost no signal in the supporting electrolyte (0.1 M KNO_3_). On the contrary, 3DG/PB-AuNPs electrode displays obvious redox peaks at 0.248 V and 0.287 V, representing the conversion between PB and PW. The peak-to-peak difference (Δ*E*_p_) is only 39 mV, demonstrating facilitated electron transfer by AuNPs. When pDA film and AuNPs formed *in situ* were modified on 3DG/PB-AuNPs electrode, the peak current increased due to the hydrophilic nature of pDA^31^ and the improved conductivity of the modified layer by AuNPs. When MPBA molecules were assembled on AuNPs, the peak current response decreased, proving successful modification of MPBA on AuNPs. After 3DG/PB-AuNPs/PDA-AuNPs/MPBA electrode was incubated with d-glucose solution, the peak current response further reduced, proving the binding of glucose.

### 3.3 Characterization of 3D non-enzymatic electrode and optimization of glucose detection

**Fig. 6:**
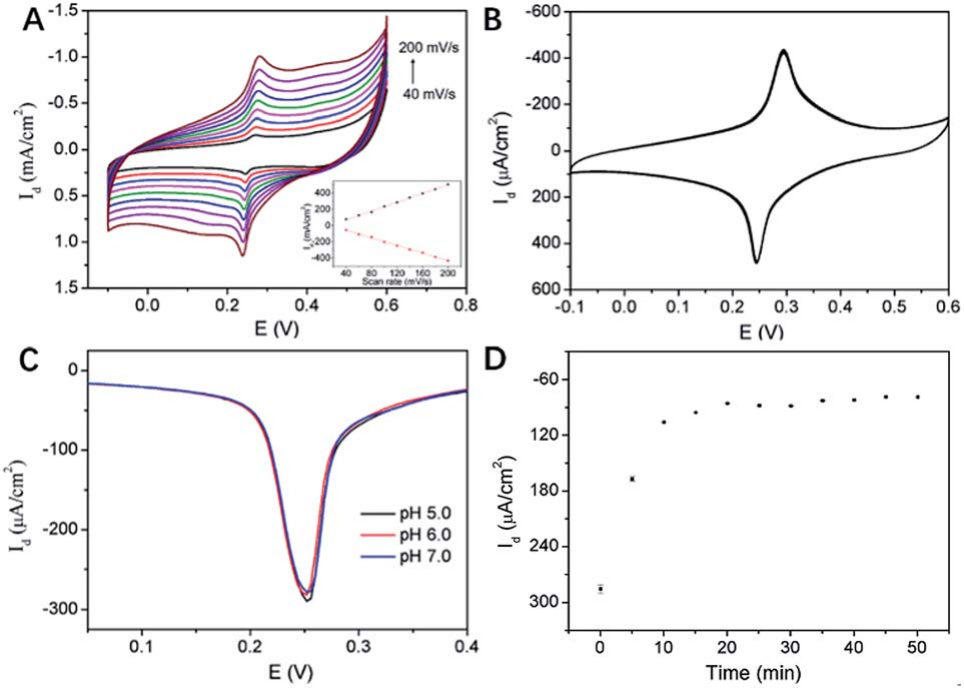
(A and B) CVs of 3DG/PB-AuNPs/PDA-AuNPs/MPBA at different scan rate (A) or in successive scans (B) in KNO_3_ solution. Inset in (A) was the anodic peak current (top) or catholic peak current (bottom) *vs.* scan rate. (C) DPV curves on 3DG/PB-AuNPs/PDA-AuNPs/MPBA at different pH. (D) DPV current density on 3DG/PB-AuNPs/PDA-AuNPs/MPBA after binding with glucose (65 μM) for different time.

Fig. 6A illustrated the CV curves of 3DG/PB-AuNPs/PDA-AuNPs/MPBA electrode at different scan rates. The anodic and cathodic peak currents of this 3D electrode were nearly symmetric, and they both linearly scaled with the scan rate, indicating a reversible and surface-controlled electrochemical process. In addition, the redox peak potential hardly changed with increasing the scan rate, demonstrating high electron transfer rate because of the supporting graphene and the introduction of abundant AuNPs. The biggest challenge for reagentless electrochemical sensors is the instability of surface-confined redox indicator. The poor stability of PB resulting from the leakage from electrode surface usually greatly hinders its practical application. To evaluate the stability of PB-AuNPs on the fabricated sensor, continuous CV scanning of 20 cycles was performed. As shown in Fig. 6B, the redox peak currents on 3DG/PB-AuNPs/PDA-AuNPs/MPBA electrode were nearly unchanged during a continuous CV scanning in PBS, indicating good stability of the immobilized PB. On the contrary, 3DG/PB-AuNPs electrode only remained 89.8% of the initial current in the continuous scanning, indicating poor stability. Thus, the protective effect of PDA film remarkably improved the stability of the surface-confined redox indicator. The pH stability of the electrode was also investigated. Slightly acidic and near-neutral detection conditions (pH 5–7) for common bioanalysis were selected. As shown in Fig. 6C, the oxidation peak current of 3DG/PB-AuNPs/PDA-AuNPs/MPBA electrode is almost unchanged, indicating a good stability of this 3D electrode in weakly acidic or neutral pH range. The optimal pH for the detection of glucose was selected as 7.0, which is also the physiological pH.

MPBA can specifically bind to glucose by forming a five-membered cycloborate.^33–35^ The binding kinetics of glucose on 3DG/PB-AuNPs/PDA-AuNPs/MPBA electrode was investigated to determine the optimal binding time. Fig. 6D gave the differential pulse voltammetry (DPV) curves of this 3D electrode after binding with glucose for different time. With increasing the incubation time, the electrochemical signal decreased and finally reached a steady state after 15 min. Thus, 15 min was selected as the optimized incubation time for further measurements.

### 3.4 Non-enzymatic and reagentless detection of glucose

**Fig. 7:**
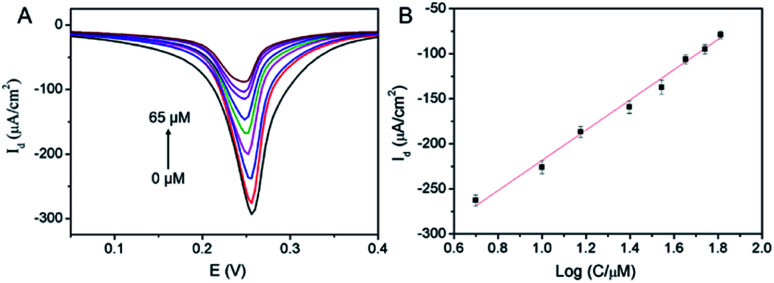
(A) DPV curves of 3DG/PB-AuNPs/PDA-AuNPs/MPBA towards different concentrations of glucose. (B) The linear relationship between the DPV peak current density and the logarithm of concentrations of glucose.

The application of 3DG/PB-AuNPs/PDA-AuNPs/MPBA electrode for non-enzymatic and reagentless detection of glucose is investigated. Owing to the high affinity between MPBA and glucose through cyclic ester bond formation,^41–44^ glucose could bind to MPBA on the sensing interface. The binding obstructs the diffusion of K^+^ across the modification layer and the electron transfer, which is reflected by the decrease in the electrochemical signal of surface-confined PB. Thus, the enzyme-free electrochemical detection of glucose could be realized by measuring the change in PB electrochemical signals before and after glucose binding. Fig. 7A demonstrated the DPV curves of 3DG/PB-AuNPs/PDA-AuNPs/MPBA electrode towards different concentration of glucose. As the concentration of glucose increases, the peak current of the electrode decreased significantly. The DPV peak current density (*I*_d_) is related to the glucose concentration. As shown in the inset of Fig. 7B, this non-enzymatic and reagentless sensor is able to determine glucose ranged from 5 μM to 65 μM (*I*_d_ = 167.7 log *C* − 386.0, *R*^2^ = 0.9929) with a detection limit (DL) of 1.5 μM (at a signal-to-noise ratio of 3). The DL value is much lower than the glucose level (usually in the range of 4.4–6.6 mM)^32^ in human blood, so the sensor might be used for glucose detection in human blood.

Comparison between non-enzymatic detection of glucose exploiting the same glucose recognition mechanism is provided in Table S1 (ESI).† The LOD is lower than those obtained using amino-PBA functionalized polymer,^44^ or MPBA-decorated gold nanoparticle-chitosan modified electrode^45^ or boronic acid-modified hydrogel coated quartz disc.^46^ The initial concentration of linear detection range of our sensor is lower than that of conductive polymer-decorated CuCo_2_O_4_ carbon nanofiber modified electrode,^47^ or glucose-imprinted pyrrole amino-PBA modified electrode,^48^ pyrene-1-boronic acid modified graphene,^49^ or MPBA monolayer on Au coated optical fiber.^50^ The LOD is higher than that obtained using amino-PBA-functionalized graphitic carbon nitride quantum dots,^51^ or boronic acid-functionalized hierarchically porous metal–organic frameworks.^52^ In comparison with other technologies (*e.g.* fluorescence, field effect transistor, surface plasmon resonance), our electrochemical sensing is attractive because of high sensitivity, rapid analysis, no need of bulky instrumentation and easy operation.

### 3.5 Stability, reproducibility, selectivity, and real-sample analysis of the developed glucose sensor

The response of this non-enzymatic and reagentless sensor to 10 μM glucose retained 92.6% after a 7 day storage. The electrode-to-electrode reproducibility was evaluated using seven electrodes prepared under the same conditions independently and an RSD of 3.0% (response to 10 μM glucose) was obtained. The high stability and good reproducibility are ascribed to the facile modification strategy, co-deposition of AuNPs and PB, and the protective effect of PDA layer.

To study the selectivity of the fabricated sensor, the effects of electroactive substances such as dopamine (DA), uric acid (UA) and ascorbic acid (AA) on glucose detection were investigated. Since the relative low concentration of DA, UA, and AA in serum (in the order of μM) in comparison with glucose ([d-glucose] ≈ 5 mM), the concentration ratio of glucose to the possible interference was set as 30 : 1. The response of 3DG/PB-AuNPs/PDA-AuNPs/MPBA electrode towards glucose was measured before and after the interference substance was added. It could be seen that no significant change was observed after adding UA, AA, or DA because of low affinity between MPBA and these biological related substrates (Fig. S5 in ESI†). In addition, the possible interference of carbohydrate on the detection of glucose was also investigated because of the similar structure with glucose. Fructose is chosen as the model carbohydrate because it is the second most abundant sugar in the human body and has preferential affinity with boronic acid moiety. However, the normal physiological concentration of fructose is approximately 200 times smaller than that of glucose.^53^ Thus, the concentration ratio of glucose to fructose was set as 100 : 1 and no significant interference was observed (Fig. S5 in ESI†).

To further prove the feasibility of our sensor for real sample analysis, three fresh human serum samples were 200-fold diluted with PBS (0.05 M, pH 7.0) before determination by the sensor. With standard addition method, 30.0 μM d-glucose was added into the diluted samples, and the determined concentration of added glucose was calculated based on the current change measured by the sensor and the working curve. The spiked experiments present satisfactory recoveries (between 95.5% to 105.3%) for the three samples, suggesting the developed sensor can be applied in real sample analysis.

## 4. Conclusions

In summary, we have developed a non-enzymatic electrochemical sensor based on functional 3D graphene for reagentless and sensitive detection of glucose. The sensor is advantageous in terms of facile preparation, high sensitivity, and good stability. Such excellent performance can be attributable to (1) large active surface area and good conductivity of 3D electrode; (2) facilitated electron transfer of PB by AuNPs; (3) improved stability of PB by protective polydopamine film. This strategy might open up a new venue for the functionalization of 3DG and might be extended for developing various highly sensitive and reagentless sensors based on 3DG for detection of a variety of analytes.

## Conflicts of interest

There are no conflicts to declare.

## Supplementary Material

RA-010-D0RA05553K-s001
