# Supplementary material for: Functional nanostructure-loaded three-dimensional graphene foam as a non-enzymatic electrochemical sensor for reagentless glucose detection
Source: RSC Adv. 2020 Sep 11;10(56):33739–46. doi: 10.1039/d0ra05553k (PMC9056722; doi:10.1039/d0ra05553k)
Supplement: RA-010-D0RA05553K-s001 [file RA-010-D0RA05553K-s001.pdf]

Electronic Supplementary Material (ESI) for RSC Advances.
This journal is © The Royal Society of Chemistry 2020

Supplementary Information

Figures

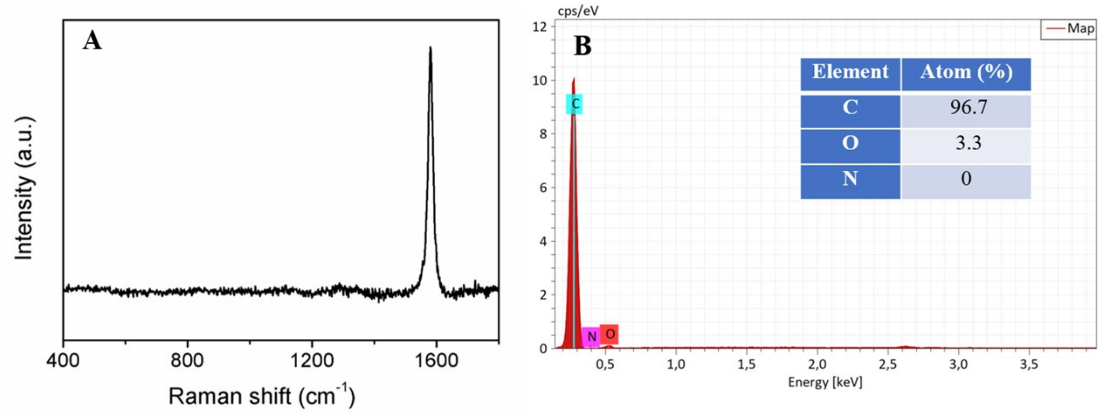

Fig. S1. Raman spectrum (A) and energy dispersive spectrum (B) of 3DG.

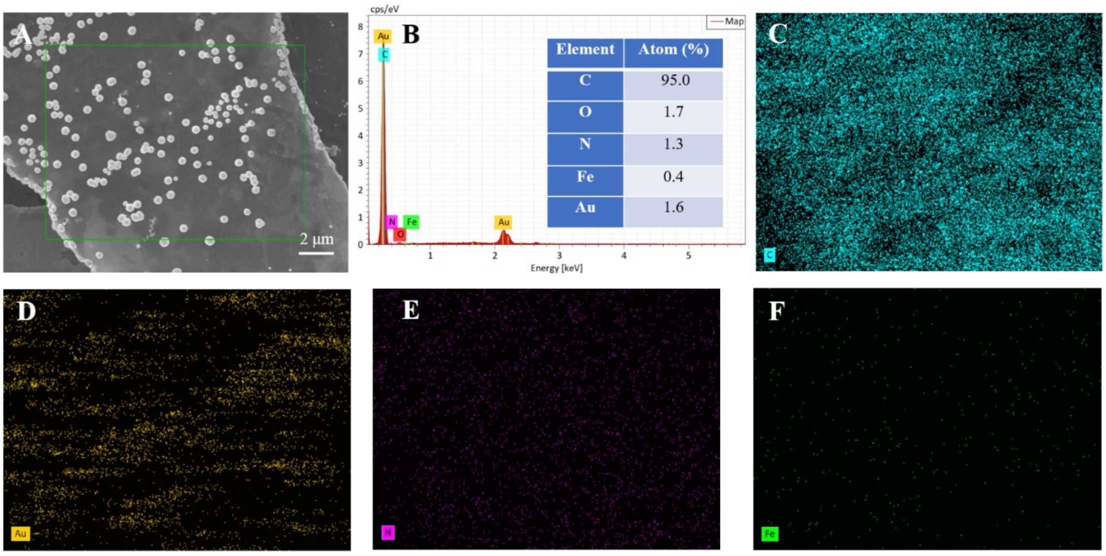

Fig. S2. SEM (A) image, energy dispersive spectrum (B) and element mapping images (C-F) of 3DG/PB-AuNPs.

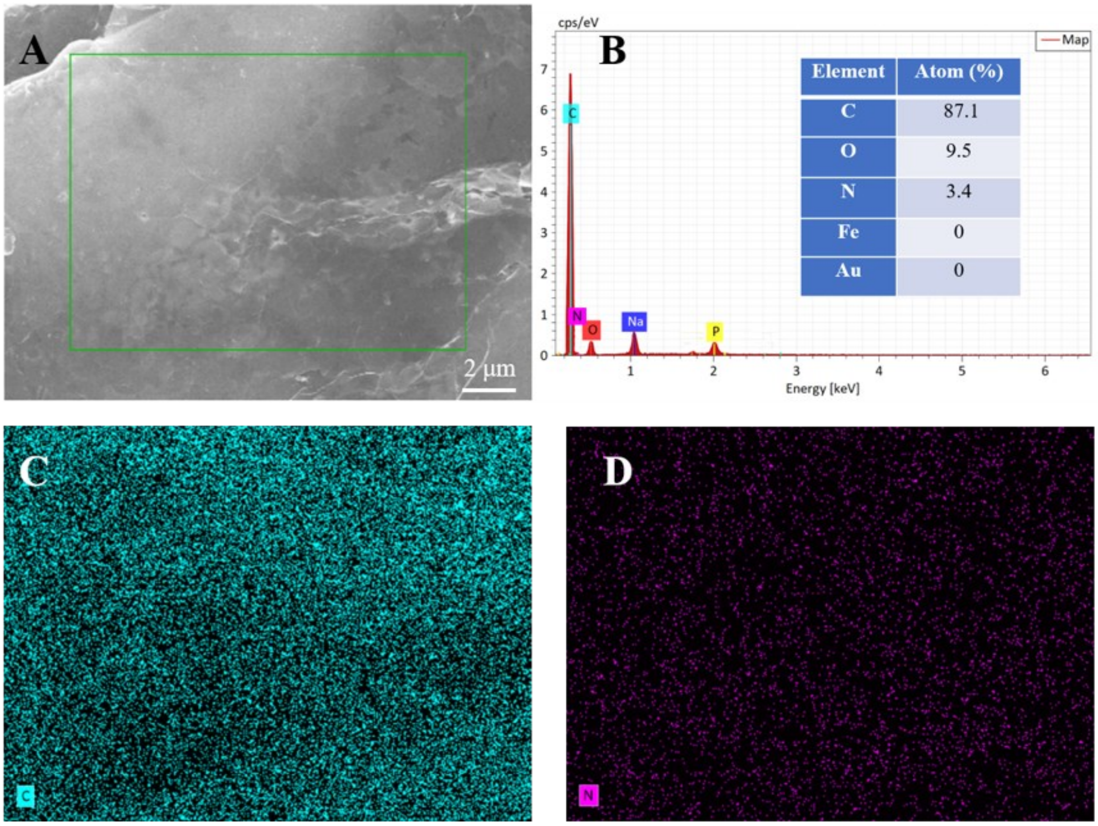

Fig. S3. SEM (A) image, energy dispersive spectrum (B) and element mapping images (C-F) of 3DG/PB-AuNPs/PDA. The signals of Na and P results from the PBS applied in DA polymerization.

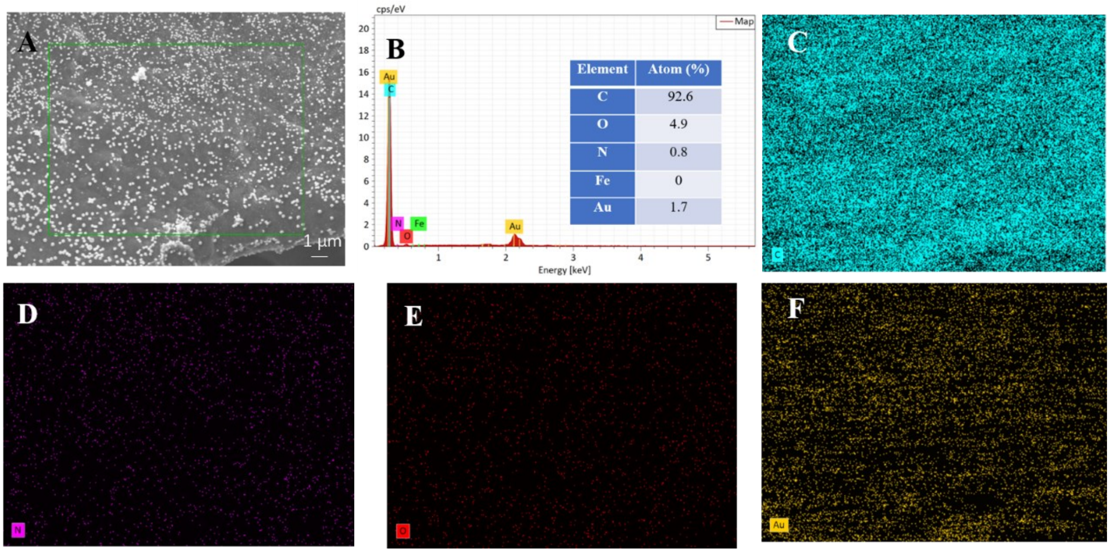

Fig. S4. SEM (A) image, energy dispersive spectrum (B) and element mapping images (C-F) of 3DG/PB-AuNPs/PDA-AuNPs.

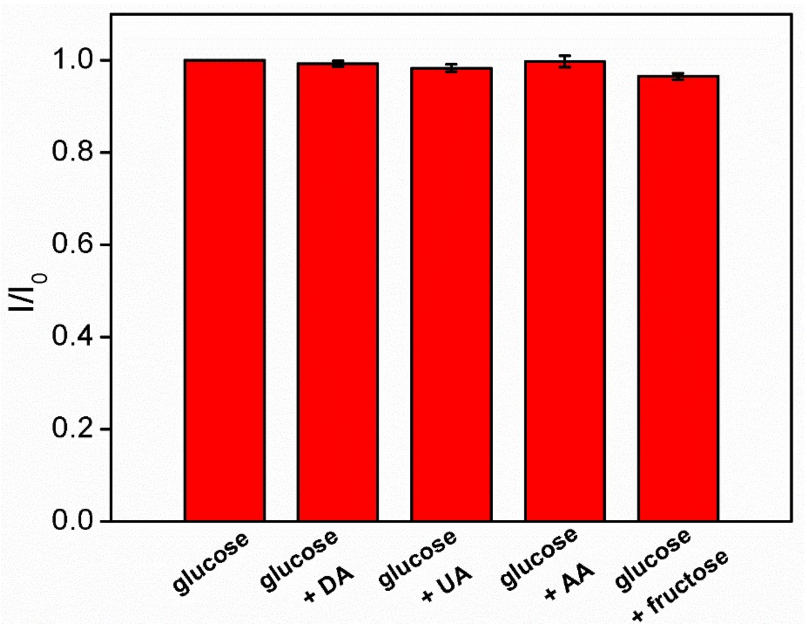

Fig. S5. The relative current ratio of 3DG/PB-AuNPs/PDA-AuNPs/MPBA towards glucose (50 μM) before (I_0) and after addition of (I) DA, UA, AA, or fructose.

Tables

Table S1 Comparison between non-enzymatic detection of glucose based on affinity between glucose and boronic acid moiety

Materials	***Method***	***detection range***	***LOD***	***Ref.***
amino-PBA-functionalized graphitic carbon nitride quantum dots	fluorescence	0 - 10 mM	42 nM	50
amino-PBA functionalized polymer	fluorescence	0.1 - 14 mM	50 μM	43
conductive polymer-decorated $CuCo_2O_4$ carbon nanofiber modified electrode	electrochemistry	0.01 - 0.5 mM, 0.5 - 1.5 mM	0.15 μM	46
MPBA-decorated gold nanoparticle-chitosan modified electrode	electrochemistry	0.5 - 30 mM	0.3 mM	44
glucose-imprinted pyrrole amino-PBA modified electrode	electrochemistry	1.1 - 44.4 mM	-	47
boronic acid-functionalized hierarchically porous metal-organic frameworks	colorimetry	2 - 100 μM	0.98 μM	51
pyrene-1-boronic acid modified graphene	field effect transistor	0.05 - 100 mM	0.15 μM	48
MPBA monolayer on Au coated optical fiber	surface plasmon resonance	0.01 - 30 mM	80 nM	49
boronic acid-modified hydrogel coated quartz disc	quartz crystal microbalance	0 - 10 mM	1 mM	45
3DG/PB-AuNPs/PDA-AuNPs/MPBA	electrochemistry	5 - 65 μM	1.5 μM	This work
